# Are different generations of female employees trapped by work-family conflicts? A study on the impact of family-supportive supervisor behavior on thriving at work

**DOI:** 10.3389/fpsyg.2024.1339899

**Published:** 2024-06-21

**Authors:** Xiao Zhao, Tian Zhang, Myeongcheol Choi, Jingyi Xu

**Affiliations:** ^1^Business School, Ludong University, Yantai, Shandong, China; ^2^Department of Business, Gachon University, Seongnam, Gyeonggi, Republic of Korea

**Keywords:** family-supportive supervisor behavior, thriving at work, work-family balance, female employees, generational differences

## Abstract

**Introduction:**

With women's advancement in education and status, they drive corporate and social progress. However, traditional gender roles burden female employees with more family responsibilities, challenging work-life balance and affecting job performance. Organizations should supporting female employees to address these challenges. Thriving at work, a core aspect of positive work engagement, helps maintain enthusiasm and efficiency. This study explores the impact of family-supportive supervisor behavior (FSSB) on the thriving at work of female employees in China, considering generational differences in their work-family balance needs and the mediating mechanisms involved.

**Methods:**

The methodology adopted in this study utilized Amos 26.0 and SPSS 25.0 to analyze data obtained from a sample of 279 female employees in China. Specifically, the study examined the direct impact of FSSB on thriving at work, alongside the mediating influence of work-family balance. Moreover, the research aimed to discern variations in these effects across different generational cohorts.

**Results:**

This study highlights the direct impact of FSSB on female employees' thriving at work across different generational cohorts. Notably, the “post-90s” generation displayed the strongest direct effect of FSSB on thriving at work. Additionally, the impact of FSSB on work-family balance varied by generation, with the “post-90s” generation showing the weakest effect. Furthermore, the mediating role of work-family balance differed among generations, with complete mediation observed in the “post-80s” generation but no mediating effect in the “post-90s” generation, reflecting their distinct work-life balance priorities and needs.

**Discussion:**

This study uses a generational difference perspective to explore the main and mediating effects of FSSB on thriving at work, enriching the theoretical research on generational differences and providing valuable insights for future research. Practically, organizations should focus on the needs of different generations while encouraging FSSB, fostering a supportive work environment and enhancing outcomes.

## 1 Introduction

In the rapidly evolving market, intense competition among businesses underscores the importance of talent. As modern women gain self-awareness, higher education, and elevated social status, their presence in the workforce grows. They focus on achieving self-worth and career success, becoming a driving force for corporate progress and socioeconomic development. However, traditional gender roles burden female employees with more family responsibilities, making work-life balance challenging. Mounting work pressure often leads to fatigue and stress, impacting their enthusiasm and job performance. Therefore, organizations should prioritize and support female employees to address these challenges.

In recent years, thriving at work has gained significant attention as a core expression of employees' positive work engagement (Walumbwa et al., 2018). It helps employees maintain enthusiasm and high efficiency, making it desirable for managers. Thriving at work refers to a psychological state where individuals experience “vitality” and “learning” simultaneously (Spreitzer et al., 2005). It reduces work burnout (Maslach, 2003; Spreitzer et al., 2012), enhances job performance (Taneva and Arnold, 2018; Walumbwa et al., 2018), improves individual health (Walumbwa et al., 2018; Clausen et al., 2022), fosters continuous self-growth and development (Paterson et al., 2014), and ultimately promotes organizational effectiveness and prosperity (Han and Wei, 2013). Most studies support its positive effects, resulting in managers and scholars focusing on helping employees in thriving. While research on its antecedents has expanded to cover individual characteristics and organizational factors (Kleine et al., 2019), there is still room for further exploration and investigation.

Work and family domains significantly impact employees' thriving at work (Spreitzer et al., 2005). Managing a good work-family relationship increases the chances of thriving at work (Zhao et al., 2017). Female employees face challenges because of multiple work and family responsibilities, making it difficult for them to balance both roles simultaneously, negatively affecting work-family balance and work performance (Chen et al., 2020). To address this issue, organizations should offer effective resources to help female employees balance work-family relationships and maintain high energy at work. Family-supportive supervisor behavior (FSSB), an informal work-family resource, has gained attention in work-family research. FSSB refers to supervisors who understand and support employees in fulfilling work-family responsibilities (Thomas and Ganster, 1995). Studies have shown that FSSB is linked to positive work outcomes, work-family outcomes, and health-related results (Crain and Stevens, 2018).

Nevertheless, the impact of FSSB on thriving at work is still not fully understood. Although psychological wellbeing has been identified as a mediator in the relationship between family-support supervisor behavior and thriving at work (Adegbite and Bawalla, 2023), additional factors may enhance its effectiveness. Therefore, for female employees seeking work-family harmony, the role of FSSB in achieving work-family balance and thriving at work is worth exploring.

With the development of occupational diversity, organizations are now witnessing a multi-generational workforce. Generations are identifiable groups of individuals who share the same birth years and have experienced key developmental stages together (Kupperschmidt, 2000). Managing employees from different generations adds complexity for managers. These employees may have diverse work values, attitudes, and behavioral patterns due to varied life experiences and societal realities. Consequently, organizations need to recognize and understand generational differences as a legitimate issue of diversity (Arsenault, 2004). Take female employees born in China in the 1980s (“post-80s”) as an example. These professionals are at a crucial stage of building families with young children and significant responsibilities for their education. The “post-80s” generation mainly comprises single children. It is also worth mentioning that this generation faces the challenge of supporting aging parents. Indeed, balancing work and family has become an important pursuit for them due to the demands of their careers and family responsibilities (Xie and Xie, 2016). However, female professionals born in the 1990s (“post-90s”) are in a stage of just starting family life, with fewer immediate family obligations. They prioritize personal development and happiness while their parents are still active and financially independent (Yang and Wu, 2021). Therefore, work-family conflicts are less apparent for “post-90s” professional women. Meanwhile, female professionals born in the 1970s (“post-70s”) have stable careers and grown-up children, resulting in relatively weaker work-family conflicts compared to the “post-80s” generation. Evidently, this suggests that different generations of female employees have varying work-family balance needs, leading to diverse demands for family-supportive resources provided by the organization. This study aims to provide empirical evidence to understand the influence of FSSB on the work prosperity of female employees from different generations and explore the differences in mediating mechanisms. Therefore, this study examines the impact of FSSB on thriving at work and the mediating role of work-family balance from a generational difference perspective. It is worth noting that empirical research on generational differences in the academic community is relatively scarce. Therefore, taking the generational differences perspective not only adds depth and richness to the research but also provides targeted organizational resources for managers based on the different age groups of female employees. This has theoretical and practical significance in reducing work pressure, maintaining work vitality, and promoting effective work status for female employees.

## 2 Theory and hypotheses

### 2.1 Theory of generations

Research on generational differences has shown rapid growth in the past decade (Weeks and Schaffert, 2019). Mannheim (1952), a German sociologist, introduced the theory of generations in 1952. This theory suggests that “generations” or “generation cohorts” are groups of people who share a common location in society and history, resulting in similar ways of thinking and behaving due to shared experiences. Building on this idea, generations are identified as groups of individuals who share the same birth years and have experienced key developmental stages together (Kupperschmidt, 2000). The differences between generations are in terms of age and their values, work attitudes, value judgments, and behavioral patterns (Dencker et al., 2008). These differences pose significant challenges for organizational management. Consequently, the study of generational differences continues to be of value and significance for exploration. However, empirical research on generations remains scarce.

When studying generational differences, it is crucial to clarify the method of categorizing generational cohorts. Common approaches include using fixed ten-year intervals based on demographics to classify different generations, such as the “post-60s,” “post-70s,” “post-80s,” “post-90s,” and “post-00s” frequently mentioned in China. Another method involves categorizing generations based on significant historical events that have influenced the social environment, including major political events, technological revolutions, or significant socioeconomic transformations (Chen and Cui, 2014). In Western countries like the United States, scholars have proposed different categorizations that are widely adopted in academia, such as the Silent Generation (1928–1945), Baby Boomers (1946–1964), Generation X (1965–1980), Millennials (1981–1996), and Generation Z (1997–2012) (Dimock, 2019). However, due to varying national development histories and contexts, there is no unified generational categorization standard in academic research. In this study, which focuses on Chinese employees, we will refer to the categorization method proposed by Chinese scholars (Shi and Guo, 2023). Accordingly, this method involves using ten-year cycles based on birth years and considering significant historical events in China that have influenced the social environment to categorize generational groups. Specifically, the “post-50s” represent the generation from the early establishment of the People's Republic of China; the “post-60s” represent the generation of socialist construction; the “post-70s” represent the generation of economic reform and opening up; the “post-80s” represent the generation affected by the one-child policy; the “post-90s” represent the generation influenced by globalization and the information age.

### 2.2 Family-supportive supervisor behavior and thriving at work

FSSB differs from general supervisor support. While general supervisor support focuses on supporting employees in their work domain, it may not extend to supporting employees in fulfilling their family responsibilities (Kossek et al., 2011). Conversely, FSSB goes beyond supporting employees in their family domain and includes supporting them in their work domain. The support in the work domain aims to enable employees to fulfill their family responsibilities effectively while ensuring the completion of work tasks and the achievement of organizational goals (Ma et al., 2016). Based on this understanding, Hammer et al. (2007, 2009) defined FSSB as the actions displayed by supervisors to support employees in fulfilling their family responsibilities, emphasizing support in the employees' family domain. They conceptualized FSSB into four dimensions: emotional support, instrumental support, role-modeling behaviors, and creative work-family management. Numerous academic studies have consistently shown that FSSB is linked to positive work-related outcomes for employees, such as increased job satisfaction (Odle-Dusseau et al., 2012; Bagger and Li, 2014; Saha, 2023), improved job performance (Odle-Dusseau, 2008; Erdogan et al., 2022; Li et al., 2023), enhanced organizational commitment (Odle-Dusseau et al., 2012; Mills et al., 2014), and higher work engagement (Matthews et al., 2014; Qing and Zhou, 2017; Rofcanin et al., 2017), among others.

As a desired state of work for managers, there has been limited research on the impact of FSSB on thriving at work. This study proposes that when employees perceive FSSB, they are more likely to get the status of thriving at work. Drawing on Spreitzer et al. (2005) socially embedded model of thriving at work, thriving at work is influenced by departmental context, work resources, and proactive behaviors. Supervisor support is a crucial resource for employees (French and Shockley, 2020). FSSB is seen as a resource based on the conservation of resources theory (Hobfoll, 1989), allowing individuals to manage work and family responsibilities, reducing stress, and improving work, family, and health outcomes (e.g., Crain et al., 2014; Yragui et al., 2017; Chambel et al., 2022).

Moreover, FSSB is considered a resource in the job demands–resources theory (Bakker and Demerouti, 2007), mitigating negative effects or improving outcomes (Crain and Stevens, 2018). Emotional support from family-supportive supervisors aligns with positive emotional resources in the socially embedded model of thriving at work (Spreitzer et al., 2005). When employees feel cared for and supported by supervisors concerning their personal lives and family needs, it enhances job satisfaction (Odle-Dusseau et al., 2012; Bagger and Li, 2014; Saha, 2023) and subjective wellbeing (Matthews et al., 2014; Wang et al., 2023). This fosters positive emotions as well as proactive behaviors and ultimately contributes to thriving at work. Therefore, we propose the following hypotheses:

*H1:* Family-supportive supervisor behavior is positively related to thriving at work.

*H2:* The effectiveness of family-supportive supervisor behavior on thriving at work is influenced by generational differences.

### 2.3 Family-supportive supervisor behavior and work-family balance

FSSB focuses on supporting employees in their family and work domains and is more effective in reducing work-family conflict than formal organizational support (Yu et al., 2022). It effectively helps employees balance work and family responsibilities (Hammer et al., 2007) and creates favorable conditions for efficient job performance (Rofcanin et al., 2017). Specifically, FSSB informally provides more flexible work hours, enhances employees' sense of control over their work, reduces work-related stress, and serves as a positive example in work-family management, all of which decrease work interference with family responsibilities (Thomas and Ganster, 1995; Hammer et al., 2009; Maloni et al., 2019). Additionally, the flexible arrangements for workplace demands and work hours enable employees to better fulfill their family responsibilities, reducing family interference with work duties (Byron, 2005; Michel et al., 2011). According to Greenhaus and Allen (2011), the decrease in work-family conflict is associated with a stronger sense of work-family balance.

Furthermore, family-supportive supervisors are adept at understanding employees' work-family needs and actively imparting experiences in balancing work and family, providing employees with additional resources (Hammer et al., 2013). According to the conservation of resources theory, employees with more resources can better navigate between their work and family domains and achieve better work-family balance (Nie and Xie, 2018). Therefore, we propose the following hypotheses:

*H3:* Family-supportive supervisor behavior is positively related to work-family balance.

*H4:* The effectiveness of family-supportive supervisor behavior on work-family balance is influenced by generational differences.

### 2.4 The mediating role of work-family balance

Supervisors serve as a potent source of assistance in helping employees successfully balance their work and family commitments (Hammer et al., 2007; Russo et al., 2018). FSSB plays a crucial role in enabling employees to perceive their supervisors' genuine support as they endeavor to strike a delicate balance between their demanding work and family commitments (Hammer et al., 2009; Kossek et al., 2011). This invaluable support not only provides emotional encouragement but also equips employees with the necessary resources to cope effectively with the inherent conflict and stress that often arise from juggling work and family responsibilities (Yu et al., 2022).

By facilitating such a harmonious balance, FSSB significantly enhances employees' capacity to transition smoothly and seamlessly between their work and non-work roles, resulting in a notable improvement in their overall work engagement levels (Rofcanin et al., 2017). As Spreitzer et al. (2005) elucidated through the lens of the socially embedded model of thriving at work, individuals are more likely to achieve thriving at work when they are fully immersed and focused in their tasks, leading to heightened vitality and ultimately paving the way for the realization of thriving in their professional lives. This positive cycle of engaged work and enhanced vitality further contributes to their ability to thrive in their work environment. Therefore, we propose the following hypotheses:

*H5:* Work-family balance mediates the relationship between family-supportive supervisor behavior and thriving at work.

*H6:* The mediation effect of work-family balance between family-supportive supervisor behavior and thriving at work is influenced by generational differences.

**Figure 1:**
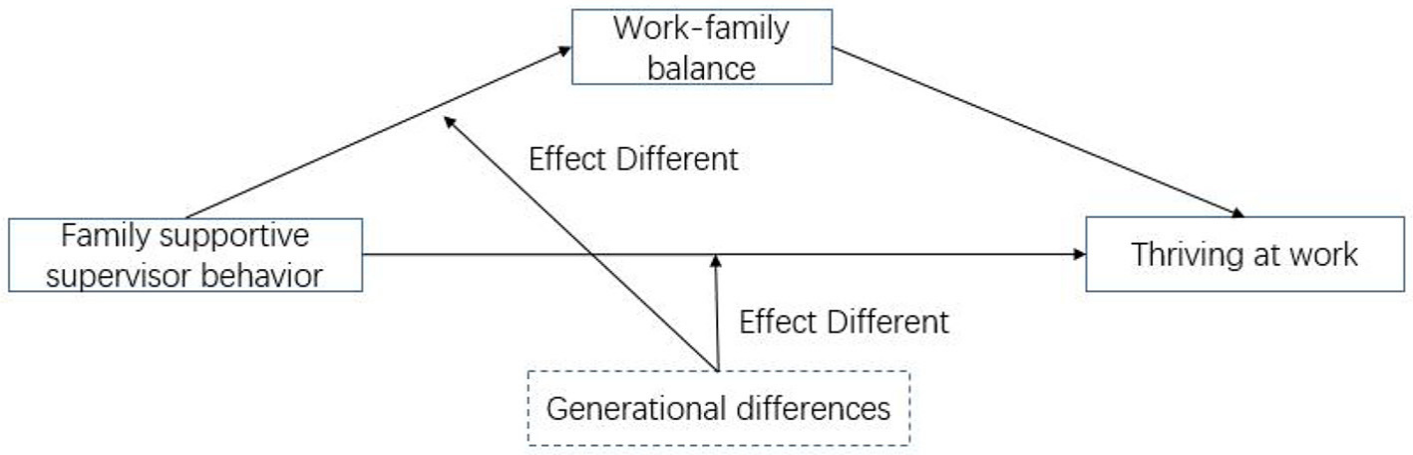
Research model.

The research model of this study is shown in Figure 1.

## 3 Methods

### 3.1 Sample and data collection

Survey data were collected from professional women residing in Beijing, Shanghai, Guangdong Province, and Shandong Province in China. Both electronic and paper questionnaires were utilized for data collection. The survey focused on married female employees employed in technology-based and manufacturing private enterprises in these regions, where the companies had over 100 employees. These female employees are primarily engaged in sales, research and development, or operations. We limited the age range of the survey participants to between 23 and 53 years, encompassing three generational groups: group “post-90s” (aged 24–33), group “post-80s” (aged 34–43), and group “post-70s” (aged 44–53). We excluded individuals from group “post-00s” (aged below 23) and group “post-60s” (aged 54 and above) because the former has just graduated from college, and the latter is nearing retirement, making their inclusion less meaningful for this study. To ensure a balanced representation of the three generational groups, we distributed the questionnaires through local universities, research institutions, and specialized survey agencies. We entrusted them to handle the distribution process and endeavor to maintain a roughly equal number of respondents from each generational group. We distributed the questionnaires through local universities, research institutions, and specialized survey agencies, instructing them to endeavor to maintain a roughly equal number of respondents from each generational group. Random sampling was then applied. To ensure the authenticity and validity of the data, we avoided using emotionally charged language, concealed the names of related variables, and designed the questions to avoid social desirability bias. Additionally, we clearly explained the purpose of the survey to the participants and assured them of the confidentiality of their responses to alleviate any concerns. In the end, we received responses from a diverse and representative sample of participants. A total of 349 out of 450 questionnaires were found to be complete in all respects, leading to a response rate of 77.6%. In order to achieve a relatively balanced sample size across groups for better comparison, we randomly selected 100 samples from each group based on generational differences. From these samples, we further collected valid samples, resulting in a final distribution of 93 samples for group “post-70s,” 96 samples for group “post-80s,” and 90 samples for group “post-90s” for each group, totaling 279 samples for group “all-sample.” A detailed sketch of the demographic properties of the respondents is given in Table 1.

**Table 1:** Respondent's profile.

**Demographic variable**	**Categories**	**Group “post-70s” (*****N =*** **93)**		**Group “post-80s” (*****N =*** **96)**		**Group “post-90s” (*****N =*** **90)**	
		**Frequency**	**%**	**Frequency**	**%**	**Frequency**	**%**
Education level	Junior college and under	35	37.6	19	19.6	7	7.7
	Undergraduate	46	49.5	48	49.5	57	62.6
	Master's and above	12	12.9	29	29.9	26	28.6
Years of work	≤ 5 years	0	0.0	4	4.1	66	72.5
	6–10 years	3	3.2	24	24.7	20	22.0
	11–15 years	22	23.7	51	52.6	4	4.4
	16–20 years	47	50.5	10	10.3	0	0.0
	>20 years	21	22.6	7	7.2	0	0.0
Position level	Junior staff	16	17.2	50	51.5	70	76.9
	Junior manager	29	31.2	25	25.8	16	17.6
	Middle manager	33	35.5	15	15.5	4	4.4
	Senior manager	15	16.1	6	6.2	0	0.0
Years of marriage	≤ 5 years	11	11.8	15	15.5	67	73.6
	>5 years	82	88.2	81	83.5	23	25.3
Number of children	0	21	22.6	26	26.8	79	86.8
	1	59	63.4	45	46.4	10	11.0
	2	13	14.0	23	23.7	1	1.1
	>3	0	0.0	2	2.1	0	0.0

### 3.2 Measures

The measurement scales employed in this study were sourced from established scales known for their validity and reliability. A double translation process was conducted to revise all scales to ensure accuracy. Respondents were asked to rate the items on a five-point Likert-type scale, ranging from 1 (strongly disagree) to 5 (strongly agree).

To assess FSSB, a short-form scale of the FSSB scale was employed. The original FSSB scale, developed by Hammer et al. (2009), consists of 14 items across four dimensions: emotional support (4 items), instrumental support (3 items), role modeling (3 items), and creative work-family management (4 items). In a subsequent study by Hammer et al. (2013), a 4-item short-form of the FSSB scale was created by selecting one representative item from each dimension. This short-form of the FSSB scale has been widely adopted due to its time efficiency in administration and established reliability and validity. A sample item is “My supervisor demonstrates effective behaviors in how to juggle work and non-work issues.”

The assessment of thriving at work utilized a ten-item scale developed by Porath et al. (2012). The scale comprises two dimensions: vitality and learning, each consisting of five items. A sample item for learning is “I find myself learning often.” A sample item for vitality is “I feel alive and vital.”

Work-family balance was assessed using a six-item scale proposed by Grzywacz and Carlson (2007). To adapt the scale to the Chinese context, appropriate revisions were made. Sample items from the scale include: “I am able to effectively negotiate and fulfill my responsibilities with important individuals in both my work and family domains, such as spouse, children, supervisors, and colleagues.”

## 4 Results

### 4.1 Common method bias

Considering that all the scales used in this study were self-report measures, Harman's single-factor test was conducted to check whether there was a significant issue of common method bias. It was found that the first factor explained 37.94% of the total variance in the group “all-sample.” Similarly, it was 38.69%, 37.37%, and 38.21% in groups “post-70s,” “post-80s,” and “post-90s,” respectively. As observed, they were all below the acceptable limit of 40%, indicating no sign of bias in all data groups.

### 4.2 Descriptive analysis, reliability, validity, and correlations

The analysis was performed with Amos 26.0 and SPSS 25.0. As shown in Table 2, the reliability of constructs in all groups were more than 0.8, the composite reliability (CR) values were more than 0.7, and the average variance extracted (AVE) values were more than 0.5. Additionally, for each variable, its square root of AVE was always higher than the correlation between itself and other variables in all data groups. Confirmatory factor analysis was conducted using Amos 26.0 to further test discriminant validity, as shown in Table 3. The results indicate that all of the fitting indices of the model of group “all-sample” meet the recommended values, which shows an acceptable model fit. Meanwhile, most of the fitting indices of other models meet the recommended values, indicating that they all have good fit and overall goodness of fit.

**Table 2:** Descriptive analysis, correlations, and discriminant validity.

**Sample**	**Variables**	**Mean**	**SD**	**Cronbach's α**	**CR**	**AVE**	**1**	**2**	**3**
Group “all-sample” (*N =* 279)	**1. FSSB**	3.545	0.736	0.827	0.802	0.504	*(0.710)*		
	**2. WT**	3.489	0.761	0.904	0.910	0.506	0.394^**^	*(0.711)*	
	**3. Ban**	3.529	0.596	0.850	0.857	0.501	0.475^**^	0.403^**^	*(0.708)*
Group “post-70s” (*N =* 93)	**1. FSSB**	3.309	0.834	0.853	0.806	0.509	*(0.714)*		
	**2. WT**	3.660	0.630	0.895	0.909	0.502	0.453^**^	*(0.708)*	
	**3. Ban**	3.504	0.681	0.894	0.878	0.546	0.565^**^	0.425^**^	*(0.739)*
Group “post-80s” (*N =* 96)	**1. FSSB**	3.492	0.782	0.849	0.833	0.555	*(0.745)*		
	**2. WT**	3.653	0.655	0.901	0.910	0.507	0.388^**^	*(0.712)*	
	**3. Ban**	3.606	0.578	0.815	0.864	0.517	0.379^**^	0.471^**^	*(0.719)*
Group “post-90s” (*N =* 90)	**1. FSSB**	3.308	0.825	0.809	0.817	0.530	*(0.728)*		
	**2. WT**	3.672	0.653	0.919	0.862	0.511	0.549^**^	*(0.715)*	
	**3. Ban**	3.474	0.513	0.775	0.925	0.555	0.457^**^	0.359^**^	*(0.745)*

^**^p < 0.01 (two-tailed); the italics numbers in parentheses at the end of each row are square roots of AVE; WT, thriving at work; Ban, work-family balance.

**Table 3:** Confirmatory factor analysis results.

**Sample**	**χ^2^**	**χ^2^ /df**	**RMSEA**	**GFI**	**CFI**	**IFI**	**TLI**
Group “all-sample”	125.749	2.466	0.073	0.930	0.944	0.945	0.928
Group “post-70s”	86.423	1.695	0.087	0.869	0.937	0.939	0.918
Group “post-80s”	83.415	1.636	0.082	0.887	0.929	0.931	0.908
Group “post-90s”	72.049	1.413	0.068	0.886	0.938	0.940	0.919

### 4.3 Hypothesis testing

This study used hierarchical regression analysis to test hypotheses. We first conducted tests on the “all-sample” group to validate hypotheses H1, H3, and H5. Next, we performed separate tests on the three subgroup samples to analyze the impact of generational differences. The results are presented in Table 4.

**Table 4:** Hierarchical regression analysis.

**Sample**	**Variables**	**Work-family balance**		**Thriving at work**		
		**Model 1**	**Model 2**	**Model 3**	**Model 4**	**Model 5**
Group “all-sample”	Education level	−0.004	0.038	0.044	0.088	0.076
	Years of work	−0.045	−0.020	−0.004	0.022	0.029
	Position level	0.082^*^	0.062^*^	0.101^*^	0.080^*^	0.060
	Years of marriage	0.106	0.180^*^	0.031	0.106	0.049
	Number of children	−0.070	−0.151^**^	0.121	0.038	0.087
	FSSB		0.406^***^		0.419^***^	0.289^***^
	Work-family balance					0.322^***^
	R^2^	0.026	0.261	0.074	0.228	0.275
	F	1.458	15.975^***^	4.388^**^	13.378^***^	14.673^***^
Group “post-70”	Education level	0.063	0.034	0.046	0.028	0.021
	Years of work	−0.040	0.003	0.004	0.031	0.030^**^
	Position level	0.172^**^	0.100^*^	0.205^***^	0.160^**^	0.140
	Years of marriage	0.274	0.170	−0.042	−0.106	−0.141
	Number of children	−0.208	−0.205	0.161	0.162	0.205^*^
	FSSB		0.433^***^		0.268^***^	0.178^*^
	Work-family balance					0.207^*^
	R^2^	0.103	0.360	0.259	0.374	0.406
	F	2.004	8.068^***^	6.086^***^	8.559^***^	8.294^***^
Group “post-80”	Education level	0.105	0.129	0.111	0.133	0.073
	Years of work	−0.024	−0.048	0.071	0.049	0.071
	Position level	0.024	0.050	0.080	0.104	0.081
	Years of marriage	0.097	−0.050	−0.045	−0.182	−0.158
	Number of children	−0.038	−0.077	0.318^***^	0.281^**^	0.317^***^
	FSSB		0.312^***^		0.291^***^	0.146
	Work-family balance					0.465^***^
	R^2^	0.021	0.182	0.209	0.318	0.455
	F	0.378	3.302^**^	4.746^**^	6.905^***^	10.509^***^
Group “post-90”	Education level	0.033	0.037	−0.083	−0.077	−0.083
	Years of work	−0.080	−0.019	−0.103	−0.007	−0.004
	Position level	0.056	0.024	−0.079	−0.130	−0.133
	Years of marriage	−0.032	−0.010	−0.159	−0.124	−0.122
	Number of children	0.108	0.157	0.204	0.281	0.255
	FSSB		0.288^***^		0.453^***^	0.406^***^
	Work-family balance					0.164
	R^2^	0.015	0.221	0.029	0.344	0.357
	F	0.247	3.918^**^	0.505	7.262^***^	6.507^***^

^***^p < 0.01, ^**^p < 0.01, ^*^p < 0.05 (two-tailed).

We first examined the main effect. Based on Model 4, it can be observed that FSSB has a significant positive effect on thriving at work in the group “all-sample” (β = 0.419, *p* < 0.001), and H1 was supported. Similarly, in the group “post-70s” (β = 0.268, *p* < 0.001), group “post-80s” (β = 0.291, *p* < 0.01), and group “post-90s” (β = 0.453, *p* < 0.001), FSSB all have a significant positive effect on thriving at work, which further prove that H1 is supported. The effectiveness of group “post-90s” (β = 0.453) is the highest, which is higher than the group “post-80s” (β = 0.291), whereas the group “post-70s” (β = 0.268) is the lowest. This shows generational differences in the relationship between FSSB and thriving at work; therefore, H2 was supported.

Model 2 was employed to test H3 and H4. The results show that FSSB significantly and positively influences work-family balance in the group “all-sample” (β = 0.406, *p* < 0.001) and all generational groups, supporting H3. Notably, there exist variations in the effectiveness of generational groups. The effectiveness of all generational groups is different. The highest effectiveness is observed in the group “post-70s” (β = 0.433, *p* < 0.001), whereas the lowest effectiveness is seen in the group “post-90s” (β = 0.288, *p* < 0.01). Group “post-80s” falls in the middle with a β coefficient of 0.312 (*p* < 0.001). These results suggest the presence of generational differences in the relationship between FSSB and work-family balance. Therefore, H4 was supported.

Regarding H5, based on Models 4 and 5 in the group “all-sample,” the inclusion of work-family balance (β = 0.322, *p* < 0.001) in Model 5 resulted in a decrease in the coefficient of thriving at work from 0.419 (*p* < 0.001) to 0.289 (*p* < 0.001), but it remained significant. This suggests that the mediating effect of work-family balance is significant, supporting H4. While testing for H6 and comparing Models 4 and 5, divergent results can be observed across the three generational groups when the work-family balance was included in Model 5. In group “post-70s,” the coefficient of thriving at work decreased from 0.268 (*p* < 0.001) to 0.178 (*p* < 0.05), while the coefficient of the work-family balance (β = 0.207, *p* < 0.05) remained significant, indicating a partial mediating role of work-family balance between FSSB and thriving at work in group “post-70s.” In group “post-80s,” the coefficient of FSSB changed significantly from being significant (β = 0.291, *p* < 0.001) in Model 4 to becoming non-significant in Model 5 (β = 0.146). Meanwhile, the coefficient of the work-family balance (β = 0.465, *p* < 0.001) remained significant, indicating a complete mediating role of work-family balance between FSSB and thriving at work in group “post-80s.” In group “post-90s,” the coefficient of thriving at work decreased from 0.453 (*p* < 0.001) to 0.406 (*p* < 0.001); however, the coefficient of the work-family balance (β = 0.164) became non-significant. This suggests that there was no mediation effect of work-family balance between FSSB and thriving at work in group “post-90s.” Summing up the above, it is obvious that the mediation effect of the work-family balance between FSSB and thriving at work is influenced by generational differences. Hence, H6 was supported.

To address the limitations of hierarchical regression analysis, a bootstrapping method with 5,000 resamples with the help of PROCESS macro by Hayes (2013) on SPSS was used to further test the mediation effect across all groups. Demographic variables, such as education level, years of work, position level, years of marriage, and number of children were controlled in the analysis. The results in Table 5 show that direct and indirect effects are significant in group “all-sample” and group “post-70s.” In group “post-80s,” the indirect effect is significant (β = 0.145, CI = [0.064, 0.260]), whereas the direct effect is not significant (β = 0.146, CI = [−0.005, 0.296]). Conversely, in group “post-90s,” the direct effect is significant (β = 0.406, CI = [0.246, 0.566]), whereas the indirect effect is not significant (β = 0.047, CI = [−0.012, 0.113]). From these results, it can be seen that work-family balance plays a partial mediating role in group “all-sample” and group “post-70s,” plays a complete mediating role in group “post-80s,” but doesn't play a mediating role in group “post-90s.” Therefore, these results further support H6.

**Table 5:** Regression results of mediation analysis.

**Sample**	**Effect**	**Coefficient**	**SE**	**95% CI**
Group “all-sample”	Direct effect	0.289	0.064	[0.164, 0.414]
	Indirect effect	0.131	0.031	[0.074, 0.196]
Group “post-70”	Direct effect	0.178	0.078	[0.023, 0.334]
	Indirect effect	0.090	0.038	[0.026, 0.174]
Group “post-80”	Direct effect	0.146	0.076	[−0.005, 0.296]
	Indirect effect	0.145	0.050	[0.064, 0.260]
Group “post-90”	Direct effect	0.406	0.080	[0.246, 0.566]
	Indirect effect	0.047	0.032	[−0.012, 0.113]

## 5 Discussion

First, this study validated the direct effect of FSSB on female employees' thriving at work. The empirical analysis results showed a significant positive impact of FSSB on the thriving at work of female employees, with this effect varying across different generations. Among them, the direct effect of FSSB on thriving at work was strongest in the “post-90s” generation (β = 0.453), followed by the “post-80s” generation (β = 0.291), and weakest in the “post-70s” generation (β = 0.268). This could be attributed to the fact that most employees from the “post-90s” generation have just entered the workforce and are in a stage of exploration and learning, where the influence of supervisors' management style and behavior is significant (Bauer and Erdogan, 2011; Sluss and Thompson, 2012). At this stage, FSSB is more likely to make employees feel cared for by their supervisors, providing emotional support that encourages them to discuss their issues and reduce stress, making them feel valued and understood. This emotional connection enhances Leader-Member Exchange, where employees feel treated as “insiders” by their leaders (Winkel and Clayton, 2010; Yin et al., 2021), creating a vibrant atmosphere of trust and respect, which is very beneficial for newcomers' positive work attitudes and behaviors (Ellis et al., 2019; Liu et al., 2021). In return, “post-90s” employees, who have not yet established a firm foothold in the workplace, will work harder in their jobs (Sluss and Thompson, 2012; Ellis et al., 2019), actively engage in learning to reciprocate the trust of their leaders (Bezuijen et al., 2010), and experience thriving at work (Li, 2015).

Compared to the “post-90s” generation, the “post-80s” generation currently faces more complex workplace pressures and multiple factors influencing thriving at work, where support from supervisors is just one of the contributing factors. Therefore, the direct effect of FSSB on thriving at work for the “post-80s” generation is relatively lower. Additionally, the “post-70s” generation, with a longer tenure in the workforce and a more stable work status within the organization, including some who are already managers themselves, experiences a weakest direct effect of supervisors' management on work status than the “post-80s” and “post-90s” generations.

Second, FSSB has a significant positive impact on the work-family balance of female employees, indicating that providing FSSB within the organization also varies with generational differences. Based on the empirical analysis data, it can be observed that the impact of FSSB on work-family balance is strongest for the “post-70s” generation (β = 0.433), followed by the “post-80s” generation (β = 0.312) and weakest for the “post-90s” generation (β = 0.288). Possible explanations for this finding are as follows: Both the “post-70s” and “post-80s” generations have elderly parents to care for as well as children to raise, creating a certain need for work-family balance. However, as stated in the introduction, the 80s generation, owing to factors, such as their age and being mostly only children, faces more numerous and complex factors influencing work-family balance than the “post-70s” generation. FSSB is just one of these factors, resulting in a lower impact on work-family balance for the “post-80s” generation. However, the “post-90s” generation is still in the stage of having just established their own families. The pressures of child education and elderly care for the “post-90s” generation are lower than those for the “post-80s” generation. Consequently, their demands for work-family balance are relatively lower, and the role of FSSB in improving work-family balance for the “post-90s” generation is not as evident.

Third, considering the influence of generational differences, this study examines the mediating role of work-family balance in the relationship between FSSB and thriving at work. The empirical results reveal that the mediating role varies among the three generational samples. For the “post-70s” generation, work-family balance partially mediates the positive impact of FSSB on thriving at work. In the “post-80s” generation, work-family balance fully mediates this relationship. However, no mediating effect is observed in the “post-90s” generation. This suggests that for the “post-80s” generation, the positive impact of FSSB on thriving at work is entirely achieved through work-family balance as a mediating variable. This finding further indicates that the “post-80s” generation has the highest demand for work-family balance among the three generations. As a result of greater work-family conflicts, the “post-80s” generation needs FSSB to directly improve work-family balance. When FSSB improves the work-family balance, it directly results in increased work vitality and learning motivation among the “post-80s” generation.

In contrast, the positive impact of FSSB on thriving at work for the “post-70s” generation is partially direct and partially mediated through work-family balance. This may be attributed to the fact that the demand for work-family balance is lower for the “post-70s” generation than the “post-80s” generation. With the implementation of FSSB, the “post-70s” generation experiences thriving at work as a result of both improved work-family balance and the perception of being cared for and valued by supervisors. As a generation known for gratitude and emotional connection, the “post-70s” generation tends to work harder and achieve thriving at work when they feel appreciated. For the “post-90s” generation, work-family balance does not act as a mediating variable. This finding is consistent with the current situation where work-family conflicts are relatively low for the “post-90s” generation. For them, the positive impact of FSSB on thriving at work is direct, as previously discussed. However, the mediating effect is not evident due to the current lower demand for work-family balance among the “post-90s” generation.

## 6 Implications

### 6.1 Theoretical contribution

FSSB and thriving at work have been recent research hotspots, but there is still a limited understanding of their relationship. This study explores the impact mechanism of FSSB on thriving at work and discusses the mediating role of work-family balance. These findings enrich the research on outcome variables of FSSB and antecedent variables of thriving at work, providing valuable theoretical insights.

The research sample focuses on female employees, conducting empirical analysis on female employees in major cities in China. The targeted and instructive conclusions are of significance. Existing research on similar topics has been relatively scarce regarding female samples. However, because female employees often have high demands on work-family balance, and the thriving at work status of male and female employees may differ, this study focuses on female employees, making a theoretical contribution to further understanding how to enhance female employees' thriving at work .

The major theoretical contribution of this research lies in its adoption of a generational difference perspective. Empirical studies on generational differences are still limited in academia in terms of the quantity and maturity of research methods. This gap hinders a deeper exploration of generational differences in China. Given that management increasingly values diverse generational groups, incorporating a generational difference perspective into management research is highly meaningful. This study uses data from three generations to explore the generational differences in main effects and mediating effects, resulting in intriguing findings. This enriches the conclusions of this study and the field, providing valuable methods and insights for future related research.

### 6.2 Practical implications

First, organizations should further encourage FSSB and take various measures to ensure its implementation. On the one hand, organizations can select employees who possess the ability to support female employees' families as supervisors and provide specialized training on family-supportive behaviors to existing supervisors. On the other hand, there should be increased emphasis and support within the organization for family-supportive behaviors. For instance, the organization can include supervisors' family-supportive behaviors in performance evaluations and establish a family-supportive corporate culture by making changes in policies and culture to create a supportive environment for families. Additionally, it is essential to improve incentive systems related to family-supportive behaviors, transforming them from informal organizational support to formal support. Through these institutional improvements, supervisors' family-supportive behaviors will be effectively rewarded and recognized for supporting female employees.

Second, organizations need to recognize that generational differences extend beyond age and primarily manifest in differences in values among different generations. These differences pose challenges for organizational management, making generational differences a critical factor that organizations must continuously consider. Therefore, organizations should provide more nuanced human resources management strategies by considering generational differences. Based on the conclusions of this study, organizations should recognize that work-family conflict and balance are significant issues for the “post-80s” generation. Besides offering more FSSB for this group, organizations should also pay continued attention to other factors that may affect their work-family balance. For example, providing comprehensive elder care and child care services, family medical assistance programs, and work-family conflict counseling services can create more favorable conditions for female employees to balance work and family life. Additionally, giving middle-aged “post-80s” female employees more learning opportunities and work challenges can stimulate their work vitality, help them find greater self-value in their work, and enable them to deal with family issues with more energy, achieving a win-win state of work-family balance and thriving at work.

As for the “post-90s” generation, organizations should focus on their rapid self-improvement needs and provide multi-faceted organizational and supervisory support, not limited to FSSB. Offering more learning opportunities, autonomy in work, and timely and effective work recognition can make the “post-90s” generation feel trusted by the organization and supervisors, resulting in higher work enthusiasm and learning motivation and faster attainment of work prosperity. Building upon the research foundation of this study, organizations should also consider generational differences in other management measures, move away from a one-size-fits-all approach, and implement targeted and specific management strategies based on the characteristics of different age groups, achieving better management outcomes.

Third, generational differences among female employees vary across countries due to cultural, social, economic, and policy factors. Understanding these differences is crucial for effective management and policy-making. This study focuses on China, where generational differences among female employees are notably pronounced. The conclusions provide valuable insights for managing female employees in other countries with significant generational differences. For example, in Japan, traditional gender roles are strong, but the shift away from lifetime employment system sees younger women seeking diverse careers, while older women prefer stability. Germany's robust social welfare system creates varying expectations; older women rely on traditional support, while younger women prioritize flexibility. In India, cultural and religious norms heavily influence older women, whereas younger women, shaped by globalization, seek career independence. In these countries, the noticeable generational differences among female employees reflect diverse needs across different generations and provide critical insights for businesses and policymakers aiming to optimize management and support the development of female employees.

## 7 Limitations and future work

This study has several limitations. Specifically, this study employed self-assessment reports from employees to collect data, inevitably leading to certain biases due to subjectivity or the presence of social expectations. In future research, measurement methods involving mutual evaluations between leaders and employees could be utilized, or supplemented with interview or observational techniques. Additionally, due to constraints in research conditions, this study only employed cross-sectional data from the same time point. Future research could employ longitudinal study methods to collect data from the research sample at different time points.

Moreover, in terms of research subjects, focusing on female employees in the workplace as the research participants while providing targeted conclusions also imposes certain limitations. Future research could conduct comparative studies between male and female employees to explore whether the research findings differ between genders. Additionally, the sample source of this study only involves some regions of China. Considering the vastness of China, female employees of the same generation in different regions may have different needs. Future research could consider conducting comparative analyses in different regions, such as comparing the northern inland areas with the southern coastal areas, to enrich the theoretical and managerial significance of the study.

Furthermore, although this study considers the direct effects of FSSB on thriving at work and the mediating effect of work-family balance from a generational difference perspective, it inevitably overlooks other potential mediating and moderating variables. Subsequent research could consider introducing additional moderating variables or replacing the mediating variables to further investigate the mechanism through which FSSB influences thriving at work and whether generational differences play a role.

Finally, as previously mentioned, generational differences are not unique to China. Noticeable generational differences may also exist among female employees in other countries such as Japan, Germany, and India. Therefore, future research could expand to include other countries, exploring generational differences among female employees in different cultural contexts. This would enrich the application scope of generational difference theories.

## Data availability statement

The raw data supporting the conclusions of this article will be made available by the authors, without undue reservation.

## Ethics statement

The studies involving humans were approved by Institutional Review Board of Gachon University. The studies were conducted in accordance with the local legislation and institutional requirements. The participants provided their written informed consent to participate in this study.

## Author contributions

XZ: Writing – original draft, Investigation, Methodology. TZ: Writing – review & editing, Resources, Validation. MC: Writing – review & editing, Conceptualization, Data curation, Supervision. JX: Writing – review & editing, Investigation, Formal analysis.
